# Cerebral microbleeds shouldn’t dictate treatment of acute stroke: a retrospective cohort study evaluating risk of intracerebral hemorrhage

**DOI:** 10.1186/s12883-018-1029-0

**Published:** 2018-03-27

**Authors:** Martin A. Chacon-Portillo, Rafael H. Llinas, Elisabeth B. Marsh

**Affiliations:** 1 Division of Congenital Heart Surgery, Texas Children’s Hospital, Michael E. DeBakey Department of Surgery, Baylor College of Medicine, Houston, Texas USA; 2 Department of Neurology, Johns Hopkins University School of Medicine, 600 N Wolfe St, Phipps 446, Baltimore, MD 21287 USA

**Keywords:** Cerebral microbleed, Intracerebral hemorrhage, Intravenous thrombolysis, Stroke, Thrombolytic therapy

## Abstract

**Background:**

Intravenous tissue plasminogen activator (IV tPA) after acute ischemic stroke carries the risk of symptomatic intracerebral hemorrhage (sICH). Cerebral microbleeds (CMBs) may indicate increased risk of hemorrhage and can be seen on magnetic resonance imaging (MRI). In this study, we examined the association between CMBs and sICH, focusing on the predictive value of their presence, burden, and location.

**Methods:**

Records from all patients presenting to two academic stroke centers with acute ischemic stroke treated with IV tPA over a 5-year period were retrospectively reviewed. Demographic, medical, and imaging variables were evaluated. The presence, number, and location (lobar vs nonlobar) of CMBs were noted on gradient echo MRI sequences obtained during the admission. Univariable and multivariable statistical models were used to determine the relationship between CMBs and hemorrhagic (symptomatic and asymptomatic) transformation.

**Results:**

Of 292 patients (mean age 62.8 years (SD 15.3), 49% African-American, 52% women), 21% (*n* = 62) had at least one CMB, 1% (*n* = 3) had > 10 CMBs, and 1% (*n* = 3) were diagnosed with probable cerebral amyloid angiopathy. After treatment, 16% (*n* = 46) developed hemorrhagic transformation, of which 6 (2%) were sICH. There was no association between CMB presence (*p* = .135) or location (*p* = .325) with sICH; however, those with a high CMB burden (> 10 CMB) were more likely to develop sICH (OR 37.8; 95% CI: 2.7–539.3; *p* = .007).

**Conclusions:**

Our findings support prior findings that a high CMB burden (> 10) in patients with acute stroke treated with IV tPA are associated with a higher risk of sICH. However, the overall rate of sICH in the presence of CMB is very low, indicating that the presence of CMBs by itself should not dictate the decision to treat with thrombolytics.

## Background

Early treatment with intravenous tissue plasminogen activator (IV tPA) (< 4.5 h from symptom onset) increases the proportion of patients who survive with a favorable outcome after ischemic stroke [1]. Though treatment is associated with better outcomes, the most feared complication is hemorrhagic transformation (HT), or bleeding into the area of ischemia when the tissue is reperfused. When the ischemic volume is large enough, this can result in a symptomatic intracerebral hemorrhage (sICH) that worsens morbidity and mortality [2, 3].

Cerebral microbleeds (CMBs) are small areas of signal void, 2–10 mm in diameter [4–6] that can be seen on gradient echo magnetic resonance imaging (MRI) sequences, mostly representing blood products commonly associated with disorders such as hypertension or cerebral amyloid angiopathy (CAA). Along with other known risk factors for hemorrhage such as age, renal disease, and stroke volume [7], CMBs may also be associated with hemorrhage risk [8, 9] .The reported prevalence of CMBs on pre-IV tPA imaging ranges from 15 to 40% [10, 11]. This is clinically relevant, as it is becoming increasingly common to perform an MRI as part of the acute work-up of ischemic stroke [12]. In addition, there is increasingly rapid electronic access to patient’s prior MR imaging that may show incidental CMBs. Understanding the associated risk of sICH may, therefore, be of significant clinical impact when considering the use of thrombolytics.

There is an existing literature on the association between CMBs and intracerebral hemorrhage with varying results [10, 13–16]. This discordance between studies may be due to the varied methodologies. Increased burden and patterns of location have been analyzed differently across studies, or not at all. Of note, higher CMB burdens, particularly in the lobar location, are more highly suggestive of CAA, as described by the Boston criteria [17], and may indicate a group of individuals at a higher risk of HT following thrombolysis. There may be other factors that are helpful in determining the likelihood of CAA over hypertension and related relative risk associated with each.

The use of thrombolytic therapy in the acute setting of ischemic stroke continues to grow and information regarding a patient’s CMB status is more commonly known. Given the lack of consensus in the literature regarding the relationship between CMBs and sICH in this study, we examine the association of CMBs with hemorrhage, taking into account presence, burden, location, and the likelihood of CAA, to predict hemorrhage in patients treated with IV tPA.

## Methods

### Patients

The Johns Hopkins Institutional Review Board approved this study. Data for all stroke patients hospitalized at two large academic stroke centers are kept in separate databases approved for both clinical research and quality assurance purposes. Within these datasets, we retrospectively reviewed the records of all patients presenting with acute ischemic stroke and treated with IV tPA between January 1, 2011, and December 31, 2015. Patients were excluded if they went to intra-arterial lysis (mechanical thrombectomy), or did not have a brain MRI during their hospital stay. Image acquisition time was within 72-h of tPA administration for 98% of patients, with a maximum of 6 days post-tPA. Demographic variables (sex, race, age); medical information (history of: hypertension, diabetes mellitus, stroke, transient ischemic attack, coronary artery disease, tobacco use, alcohol use, atrial fibrillation, congestive heart failure, hyperlipidemia, chronic kidney disease, liver disease, cancer; admission systolic blood pressure, serum glucose and creatinine); stroke characteristics (admission National Institutes of Health Stroke Scale (NIHSS) score [2], etiology as defined by the Trial of ORG 10172 in Acute Stroke Treatment (TOAST) criteria [18], degree of white matter disease as defined by the Cardiovascular Health Study (CHS) score [19], imaging characteristics including CMB analysis as below; and outcomes (discharge and 90-day NIHSS and modified Rankin Score (mRS)) were collected [2, 20].

### Imaging methods and variable definitions

As part of our stroke protocol, all patients underwent MRI of the brain including diffusion-, T1-, and T2-weighted sequences. All but 21 (7%) patients had susceptibility weighted gradient echo sequences (SWI) for CMB analysis (slice thickness: 5 mm, TR: 27, TE: 20, flip angle: 15). MRI examinations were performed using 3 Tesla MRI scanners (Siemens, PA). A trained investigator (MACP) reviewed all MRIs and a subset of cases was reviewed by a vascular neurologist (EBM) with an excellent inter-rater reliability (kappa = 0.87). Investigators reviewed differing values jointly and a consensus was reached.

#### Cerebral microbleed characterization

CMBs were identified as areas of signal void 2–10 mm in diameter on gradient echo sequences with associated blooming [5, 6]. Mimics (i.e. vessels, mineralization of the globus pallidus and dentate nuclei, air-borne interfaces, hemorrhages within the area of infarction, old deep intracerebral hemorrhages) were differentiated from “definite CMBs” using the Microbleed Anatomical Rating Scale [5, 6]. The distribution of the CMBs was described as lobar, deep, and infratentorial per the previously mentioned guideline [5]. CMBs located in deep and infratentorial structures were combined as “nonlobar” for CMB location analysis. Patients with CMBs in both locations were classified as having a mixed distribution. CMB burden was defined as mild-moderate (1–10 CMBs) and high (> 10 CMBs). Patients with no CMBs on imaging were used as a reference group for the analyses. Patients were defined as having probable CAA by the primary team due to clinical/imaging characteristics based on the Boston criteria [17].

#### Ischemic burden

Stroke volume was estimated on diffusion-weighted imaging using the formula: [(length) (width) (number of slices) (slice thickness)]/2 [21]. Infarct location was divided into anterior, posterior, or mixed circulations. Lesions were further classified depending on the depth of the stroke as: lobar (cortex and subcortical white matter of the frontal, parietal, temporal, occipital and insular lobes), deep (basal ganglia, thalamus, internal capsule, external capsule, corpus callosum, deep and periventricular white matter- typically due to lacunar disease) and mixed (usually large vessel occlusions involving an entire vascular territory). The degree of white matter disease was also divided into 9 categories of increasing severity using the CHS score [19].

#### Hemorrhagic transformation

Any HT after IV tPA was identified by the presence of a lesion on gradient echo MRI sequences or hyperdensity on non-contrast CT within the first 24 h post-thrombolysis [2, 22], and their radiologic classification was defined per the European Cooperative Acute Ischemic Stroke Study II [3]. sICH occurrence was determined by the primary team, as defined by the National Institute of Neurological Disorders and Stroke r-tPA Stroke Study Group [2, 22].

### Statistical analysis

Statistical analyses were performed using Stata version 13.0. Continuous variables were described using mean ± standard deviation (SD), or median (interquartile range) and analyzed through univariable comparisons using Student’s t-tests or Mann-Whitney U test for independent variables, as appropriate. Categorical variables were described as percentages and analyzed using Pearson’s chi-square or Fisher’s exact analyses. Logistic regression was used to determine the influence of multiple risk factors. Variables significant in the univariable analysis and those of particular clinical interest were included in the multivariable modeling, with a *p*-value of 0.05 considered statistically significant.

## Results

### Patient characteristics

Over a 5-year period, 365 patients were identified as having been treated with IV tPA for acute stroke at our two centers. Of these 365 patients, 45 did not have an MRI, 22 underwent intra-arterial therapy, and 6 had been wrongly classified, leaving 292 for further analysis. The mean age of the cohort was 63 years (SD 15), 49% (*n* = 142) were black, and 52% (*n* = 151) were women. The mean initial NIHSS score was 8 (SD 5.5). The mean stroke volume was 15cm^3^ (SD 38). The mean CHS score was 3 (SD 1.7). Following administration of IV tPA, there was no evidence of diffusion restriction in 30% (*n* = 87) of patients. By TOAST criteria [18], 12% (*n* = 36) were due to small vessel disease, 23% (*n* = 68) large vessel disease, 29% (*n* = 83) cardioembolic, and 36% (*n* = 105) due to other or undetermined etiology. From the 205 patients with a visible stroke, a purely cortical stroke was found in 18% (*n* = 37), deep lacune in 38% (*n* = 77), and 44% (*n* = 91) had a larger stroke involving both cortical and deep structures. Anterior circulation infarcts occurred in 77% (*n* = 157), posterior circulation strokes in 19% (*n* = 39), and 4% (n = 9) had both distributions involved.

Some form of HT occurred in 16% (*n* = 46) of our cohort, though only 2% (*n* = 6) were defined as symptomatic. Of these 46 cases, 45 (98%) occurred within the area of ischemia including 5 sICH cases; 3 (7%) were parenchyma hematoma type 1 (PH1), 2 (4%) were parenchymal hematoma type 2 (PH2), 28 (61%) were hemorrhagic infarct type 1 (HI1), and 13 (28%) were hemorrhagic infarct type 2 (HI2). Sixty-two patients (21%) had at least one CMB on post-treatment MRI. The distribution of the CMBs was: 11% (*n* = 7) purely lobar, 57% (*n* = 35) purely nonlobar and 32% (*n* = 20) mixed distribution. The majority, 95% (*n* = 59), had 1 to 10 CMBs, while only 5% (*n* = 3) had > 10 CMBs. Of the CAA cases, 67% (*n* = 2) had a high CMB burden and 33% (*n* = 1) had a mild-moderate CMB burden [17]. These CAA patients did not have a prior history of lobar intracerebral hemorrhage or a diagnosis of CAA prior to receiving IV tPA.

### Factors associated with hemorrhage

Factors associated with HT and sICH occurrence are shown in Table 1. Advanced age (*p* = .006), black race (*p* = .041), large vessel occlusion (*p* = .006), higher NIHSS scores (at admission *p* < .001, at discharge *p* = .016), elevated discharge mRS score (*p* = .032), larger stroke volumes (*p* = .001), anterior circulation stroke location (*p* < .001), CAA (*p* = .019), lower platelet counts (*p* = .039), and atrial fibrillation (*p* < .001) were associated with HT. After adjusting for covariates, higher initial NIHSS (OR 1.07; 95% CI 1.002–1.15; *p* = .044), lower platelet counts (OR 0.99; 95% CI 0.99–0.999; *p* = .038) and large vessel disease (OR 2.38; 95% CI 1.10–5.14; *p* = .027) remained significant predictors of HT (Table 2). After univariable analysis, the only predictor of sICH was a high CMB burden (*p* < .001).

**Table 1:** Factors associated with hemorrhagic transformation and symptomatic intracerebral hemorrhage

	All *n* = 292	No HT *n* = 246	HT *n* = 46	*P* value	No sICH *n* = 286	sICH *n* = 6	*P* value
Demographics							
Age, years	63 ± 15	62 ± 15	68 ± 15	**.006**	63 (51–75)	61 (59–65)	.944
Female, % (n)	52 (151)	50 (124)	59 (27)	.302	52 (148)	50 (3)	.932
Black, % (n)	49 (142)	51 (126)	35 (16)	**.041**	48 (138)	67 (4)	.437
LVD, % (n)	23 (68)	20 (50)	39 (18)	**.006**	23 (66)	33 (2)	.891
Clinical Scales							
Initial NIHSS	7.9 ± 5.5	7.4 ± 5.3	11 ± 5.7	**< .001**	2 (4–10)	11 (6–12)	.373
Discharge NIHSS	3.7 ± 5.1	3.2 ± 4.6	5.7 ± 6.7	**.016**	2 (0–4)	2.5 (1–9)	.373
Follow-up NIHSS	1.3 ± 2.6	1.2 ± 2.4	1.9 ± 3.7	.331	0 (0–2)	0 (0–2)	.994
Discharge mRS	2.6 ± 1.6	2.5 ± 1.6	3.1 ± 1.6	**.032**	2 (1–4)	3 (2–5)	.363
Follow-up mRS	1.5 ± 1.6	1.5 ± 1.5	1.8 ± 1.7	.338	1 (0–3)	2 (0–4)	.755
Imaging Parameters							
Stroke Size, mililiters	15 ± 38	11 ± 35	33 ± 46	**< .001**	1 (0–9)	19 (3–42)	.050
CHS Score	2 (2–3)	2 (2–3)	3 (2–3)	.143	2 (2–3)	2.5 (2–3)	.911
Anterior Circulation, % (n)	54 (157)	48 (117)	87 (40)	**< .001**	53 (151)	100 (6)	.218
Clinical Parameters							
SBP, mmHg	157 ± 30	157 ± 31	155 ± 24	.731	153 (136–172)	153 (145–189)	.437
Glucose, mg/dL	135 ± 71	133 ± 70	150 ± 76	.136	113 (97–148)	111 (103–116)	.924
Cr, mg/dL	1.2 ± 0.7	1.2 ± 0.7	1.1 ± 0.4	.834	1 (0.8–1.3)	1.1 (0.8–1.3)	.893
Platelets, K/cu mm	241 ± 74	244 ± 74	220 ± 66	**.039**	237 (191–276)	232 (217–253)	.957
Risk Factors							
CAA, % (n)	1 (3)	1 (1)	4 (2)	.073	1 (2)	17 (1)	.064
HTN, % (n)	79 (230)	80 (194)	78 (36)	.848	79 (225)	83 (5)	.999
DM, % (n)	27 (77)	27 (65)	26 (12)	.938	26 (75)	33 (2)	.658
Tobacco, % (n)	29 (85)	29 (72)	28 (13)	.893	29 (82)	50 (3)	.473
Alcohol, % (n)	24 (70)	26 (62)	17 (8)	.498	24 (68)	33 (2)	.725
Afib, % (n)	20 (58)	16 (40)	39 (18)	**< .001**	19 (55)	50 (3)	.098
Stroke History, % (n)	22 (63)	22 (54)	20 (9)	.884	21 (61)	33 (2)	.801
HLD, % (n)	51 (149)	51 (126)	50 (23)	.859	51 (146)	50 (3)	.999

*Continuous data is described as mean ± standard deviation or as median (interquartile range), as appropriate per its distribution*

*Afib* atrial fibrillation, *CAA* cerebral amyloid angiopathy, *CHS* Cardiovascular Health Study criteria, *Cr* creatinine, *DM* diabetes mellitus, *HLD* hyperlipidemia, *HT* hemorrhagic transformation, *HTN* hypertension, *LVD* large vessel disease etiology, *mRS* modified Rankin Score, *NIHSS* National Institute of Health Stroke Scale, *sICH* symptomatic intracerebral hemorrhage, *SBP* systolic blood pressure, *SD* standard deviation

Bold data indicates statistical significant (*p*<.05)

**Table 2:** Multivariate logistic regression for risk factors for hemorrhagic transformation

	Hemorrhagic Transformation	
	OR (95% CI)	*P*-value
Age, per 1-y increase	1.01 (0.98–1.03)	.577
Black Race	0.56 (0.27–1.17)	.121
Initial NIHSS	1.07 (1.002–1.15)	**.044**
Stroke Size	1.00 (1.00–1.01)	.509
Platelet Count	0.99 (0.99–0.999)	**.038**
Stroke Location	1.60 (0.95–2.68)	.075
CAA	5.67 (0.45–71.97)	.181
Afib	1.66 (0.66–4.16)	.277
LVD	2.38 (1.10–5.14)	**.027**

*Afib* atrial fibrillation, *CAA* cerebral amyloid angiopathy, *NIHSS* National Institute of Health Stroke Scale, *LVD* large vessel disease

Bold data indicates statistical significant (*p*<.05)

### Factors associated with CMBs

Advanced age (*p* = .011), higher CHS scores (*p* < .001), and elevated baseline systolic blood pressure (*p* = .046) were associated with the presence of CMB. Advanced age was also associated with a higher CMB burden (0 CMBs: mean age 61.57 (SD 15.93), 1–10 CMBs: mean age 67.36 (SD 12.12), > 10 CMBs: mean age 68.67 (SD 9.07); *p* = .028).

### Role of CMB in HT and sICH

A test for independence was performed between HT and sICH with the three variables of interest: CMB presence, burden, and location, shown in Table 3. High CMB burden was most significantly associated with sICH (OR 37.83; 95% CI; 2.65–539.25; *p* = .007). Of note, 1 of 3 patients diagnosed with CAA experienced a sICH, and a second patient experienced an asymptomatic hemorrhage.

**Table 3:** Cerebral microbleed location, burden, and presence stratified by hemorrhage type

	No CMB (*n* = 209)	CMB (n = 62)	*p* value	> 10 CMBs (*n* = 3)	1–10 (*n* = 59)	*p* value	Nonlobar (*n* = 35)	Lobar (*n* = 7)	Mixed (*n* = 20)	*p* value
No HT, n (%)	175 (84)	50 (81)	.707	2 (67)	48 (81)	.508	29 (83)	7 (100)	14 (70)	.309
HT, n (%)	34 (16)	12 (19)		1 (33)	11(19)		6 (17)	0 (0)	6 (30)	
No sl CH, n (%)	206 (99)	59 (95)	.567	2 (67)	57 (97)	.023	33 (94)	7 (100)	19 (95)	.189
sl CH n (%)	3 (1)	3 (5)		1 (33)	2 (3)		2 (6)	0 (0)	1 (5)	

*HT* hemorrhagic transformation, *sICH* symptomatic intracerebral hemorrhage

### Long-term outcomes

A total of 219/292 (75%) patients had a follow-up mRS at a median of 90 days (45–160 days) from symptom onset. From these, 156/219 (71%) had a mRS < 2 at follow-up and 63/219 (29%) had a mRS > 2. CMB presence, was comparable between both groups of patients, 35/156 (24%) versus 16/58 (28%) (*p* = .739).

## Discussion

This study was designed to evaluate the relationship between the presence, burden, and location of CMBs and HT following treatment with IV tPA for acute ischemic stroke. Our sICH rate was low at only 2%, but comparable to what has been reported in a prior multicenter stroke thrombolysis register of 7% [7] and a national registry of 5% [23]. Consistent with prior studies, we found that older patients, with larger [24], more clinically severe strokes [11, 25], lower platelet counts [26, 27], and a history of atrial fibrillation [11] were more likely to develop any HT; while a high CMB burden (> 10 CMBs) was the only predictor of sICH.

Looking specifically at the relationship between CMB and hemorrhage, we found an association between high CMB burden and sICH that is consistent with previously published studies. The 10 CMBs threshold was chosen based on the methodology of two recent meta-analyses and a recent multicenter trial [14, 15, 28]. Rates of sICH were comparable across studies: those without CMBs 2% [15] (ours: 1%), mild-moderate burden 6% [15] (ours: 3%), and high burden 29–50% [14, 15] (ours: 33%). One possible explanation is that these individuals had CAA, putting them at higher risk for hemorrhage due to their underlying vascular pathology.

The advantage of our smaller study was the ability to explore the association between patterns of CMB and the diagnosis of CAA, on hemorrhage risk in a population, while determining the absolute risk within a stroke cohort of similar size and composition to many urban hospital settings. Most prior meta-analyses did not take the diagnosis of CAA into account within their analyses. In our study, only 3 patients were diagnosed with CAA, but of these, one (33%) had a hemorrhage that was symptomatic. Higher numbers of CMBs may indicate a higher propensity for the breakdown of the blood brain barrier, or “leaky vessels” that may also predispose to greater risk of HT. A recent meta-analysis including CAA in its analysis supports an association between greater numbers of CMB and increased rates of HT [29].

In our population, 21% of patients had at least one CMB present on imaging, consistent with previously published rates. While this observation might suggest that we should screen for the presence of microhemorrhages when considering treatment, it is also important to point out that CMB presence alone was not associated with either HT or sICH, and that the number of patients with > 10 CMB was very low (*n* = 3, 1%), consistent with prior cohorts [15]. Additionally, despite a CMB prevalence of 1 in 5, our rate of sICH rate was very low. These results suggest that the presence of CMBs by itself should not be considered a predictor for sICH and that MRI in the acute setting to evaluate for their existence is likely unnecessary given low rates of numerous CMBs.

Our findings are consistent with many prior studies; [10, 16] however, three recent large meta-analyses [13, 15, 29] do report CMB presence as an independent risk factor for HT. These meta-analyses were large, and predominantly used univariable modeling to evaluate a relationship between HT and only the presence of microbleeds. Data were described mainly in aggregate, with wide confidence intervals, as they were unable to account for population characteristics varying by center [30]. Charidimou and colleagues did evaluate CMB burden and found, similar to our study, that larger numbers were associated with increasing hemorrhage risk and poorer outcome [29]. Along with our study, these studies all emphasize that larger numbers of microbleeds, in many cases suggestive of CAA, is associated with HT, but that the rate of hemorrhage after tPA is low enough that a very large number of patients must to be treated to see the effect, calling into question the true clinical significance.

We also considered the importance of the location of CMBs with respect to the likelihood of HT. Our findings indicate that location is not independently associated with either symptomatic or any HT. Though this finding might also be secondary to our sample size, previous groups have had similar results [11, 15, 16, 28].

The importance of determining the association between CMB and HT after treatment with IV tPA is increasing. Historically, non-contrast head CT has been preferred over MRI in the work-up of acute stroke due to increased availability, lower cost, and more rapid time acquisition [31]. However, the use of MRI as part of the acute stroke work-up, as well as the number of patients who have had a previous MRI for another clinical indication and whose CMB status is known, is growing [12]. Understanding the relationship between CMB and HT is, therefore, critical. We have shown that individuals with a high CMB burden do have an increased risk of sICH after treatment with IV tPA; however, that the absolute risk is quite low and in most cases should not preclude treatment. While risk quantification provides clinicians with the data to make informed decisions regarding the risk/benefit ratio, other variables such as stroke size and severity, and age of the patient should continue to be the key factors driving treatment decisions [32, 33].

Our study is not without limitations. Our sample size was relatively small, potentially limiting the strength of our results due to a lack of statistical power rather than lack of an association; and from two academic tertiary referral centers in an urban setting, a distinct population. However, our population is similar to many stroke centers across the United States; and, despite a small number of sICH events, our cohort allows real life clinical application of the post-thrombolytic hemorrhage risk, both symptomatic and asymptomatic, associated with CMBs to centers of similar or smaller size and composition. In addition, for the majority of cases, imaging sequences were obtained post-IV tPA, which does not differentiate between CMBs present before IV tPA and those which appear afterward. Post-IV tPA MRIs may not be the ideal method to document CMBs due to the fact that new CMBs have been documented to occur during the first 7 days after acute ischemic stroke symptom onset in 5–13% of patients [34–36]. However, considering that for our study that would mean an additional 14–38 individuals did not have CMB on admission, we feel that our results nicely illustrate both the relatively low rate of CMB on presentation, *and* more importantly the low risk of sICH regardless of microbleed status, rendering screening prior to treatment an unnecessary use of both time and resources.

## Conclusion

Our results indicate that the overall risk of sICH following IV tPA is low, even in the presence of CMBs, which are not uncommon. Given a relative increased risk, a CMB burden of > 10 may be taken into consideration when stratifying the risk for sICH, but that even then the relative risk increase should be carefully weighed against the risks of not treating given the low absolute risk.

## Abbreviations

CAA: Cerebral amyloid angiopathy
CHSC: Ardiovascular health study
CMBs: Cerebral microbleeds
HT: Hemorrhagic transformation
IV tPA: Intravenous tissue plasminogen activator
mRS: Modified rankin score
NIHSS: National institutes of health stroke scale
SD: Standard deviation
sICH: Symptomatic intracerebral hemorrhage
TOAST: Trial of ORG 10172 in acute stroke treatment
